# Conservation of vegetable genetic diversity in Transylvania-Romania

**DOI:** 10.1038/s41598-020-75413-x

**Published:** 2020-10-28

**Authors:** Aurel Maxim, Silvia Străjeru, Cristian Albu, Mignon Sandor, Lucia Mihalescu, Sînziana Ecaterina Pauliuc

**Affiliations:** 1 Department of Environmental and Plant Protection, University of Agricultural Sciences and Veterinary Medicine, 400372 Cluj-Napoca, Romania; 2 Genebank, 720224 Suceava, Romania; 3 Department of Chemistry and Biology, North University Center of Baia Mare, Technical University of Cluj-Napoca, 430122 Cluj-Napoca, Romania

**Keywords:** Agroecology, Biodiversity

## Abstract

The conservation of plant and animal genetic heritage is not a purpose in itself, but it represents the *sine qua non* condition for practicing a sustainable agriculture and to ensure nutrition and food security on long-term. Our research focused on identifying the areas with the richest genetic diversity of vegetables in Transylvania, Romania, as well as the main vulnerabilities related to seed production for the local vegetables. Our trips included 210 locations where 338 small seed producers were surveyed. The questionnaire method with fixed questions and undisguised multiple-choices was used. A number of 316 out of 565 cultivars taken into study have been proven to be authentic and valuable landraces, meaning 55.9%. In Transylvania, the richest genetic diversity of vegetables is found in the counties of Maramures, Bistrita-Nasaud and Hunedoara—where the cooperativization was lower before the year 1989. The most important risk in losing vegetable landraces is the old age of small growers (68.4%). However, it is encouraging that many NGOs interested in identifying, conserving and promoting local varieties have emerged in the last decade. Therefore, so-called "seed houses" have been set up to facilitate the exchange of seeds, and on the other hand, the expansion of organic farming requires local varieties that are better adapted to harsh environmental conditions.

## Introduction

Term of "Landrace" has not a general accepted definition across the globe. According to Directive 2008/62/EC (rule 2), *‘landrace’ means a set of populations or clones of a plant species which are naturally adapted to the environmental conditions of their region* (27). Negri et al.^1^ and Polegri and Negri^2^ states that landraces are those variables and distinct populations that usually have a local name. Depending on the area, landraces are also called "conservation varieties", "farmer varieties", "local varieties", "primitive varieties", "local populations", "peasant varieties", "traditional varieties"^3^.

The landraces are characterised by high heterogeneity. They have the advantage of being much better adapted to condition of biotic and abiotic stress, diseases, pests, drought, low nutrient content in the soil, and of having excellent taste qualities, which can justify a higher price compared to commercial varieties^4,5^. Given these qualities, landraces are best suited for ecological systems and for sustainable ones in general, where the need for energy inputs is low.

Following the conditions of abiotic stress, generated by global warming, FAO recommends the cultivation of varieties that efficiently use water and nutrients, being tolerant to heat and pest attack. In order to obtain such varieties, breeder has to use local varieties originating in adverse environments^6,7^.

Local populations, in general, constitute an invaluable genetic potential to obtain new varieties of plants^8,9^. Their cultivation also may contribute to the development of local economies and to strengthen local cultural identity, especially if combined with agritourism^3,10–13^. In the European Union, the top valorisation of local varieties can be done through traditional agricultural products certification^14^.

The level of the genetic erosion of crop plants could not be quantified precisely because there is no complete inventory of landraces. According to Lorenzetti et al.^15^ evaluating the risk of genetic erosion is impossible in the absence of an inventory of conservation varieties. In any case, the prospect that Thuiller et al.^16^ is offering to us is worrying: 27–42% of the species of wild plants with importance for agriculture will disappear by the year 2020 as a result of global climate change. No such recent papers have been found to confirm or disprove these predictions.

In recent decades, researchers have identified the main causes of genetic erosion of vegetables, eg ageing of the population that practice traditional agriculture; replacing traditional varieties with modern varieties encouraged by the legislation of seed certification; the lack of financial support and knowledge of breeding and conservation of plants^4,13,17–20^.

The main advantage of conserving local varieties on the farm or in the home gardens is the continuation of the evolutionary process, allowing the plants to adapt to the ever-changing environmental conditions. Also, most of the times the on-farm management involves the use of a sustainable agricultural system, environmentally friendly by to the lack of application of chemical synthesis products in fertilization or control of diseases and pests^1,21^.

Ex situ conservation of agrobiodiversity is done mainly through gene banks. Lately, more and more rural communities and NGOs are collecting seeds and storing them in the so-called 'Village Seed Banks'. These seed houses have a relatively simple structure and do not involve expenditure on freezers and refrigerators as gene banks^13,22^.

In Romania, crop plant diversity is preserved by combining elements belonging to the two strategies of conservation ex situ and in situ, with a complementarity role, each having distinct advantages and disadvantages.

The purpose of our research was to make a radiography of landraces situation in Transylvania in terms of their presence, their association with traditional cultivation technologies and the risks to which they face. All researchers addressing this issue are of the opinion that the genetic erosion of crop plants cannot be stopped without knowing these fundamental aspects, regardless of the good intentions of international treaties, European directives and national laws. Secondly, our goal is for the most valuable landraces to be tested and registered in the Official Catalogue of Varieties.

## Material and methods

### Investigated areas

Our investigations were conducted in 15 of the 16 counties of Transylvania, which is the area with the greatest genetic diversity of vegetables in Romania, and also in Bucovina situated near Transylvania cf. Străjeru et al.)^23^.

Target zones and localities in each county were established on the recommendation of the county agricultural directorates that have the most complete and reliable information on all aspects of the agriculture of the respective county. At the level of locality, the guidance was received from the agricultural engineers working in the Village Hall who know very well the history and farmers’ ocuppations in each village.

The most explored areas did not pass through a cooperativization process, that were located high in the hills and mountains of lower altitude. We must note that in Communist Romania (1945–1989), the most favourable agricultural areas, suitable for industrialized agriculture, were owned by the State, forcefully, as in other communist countries. Non-cooperativizate areas are those where the farmhouses have continued to develop privately. Here, peasants continued to maintain the old varieties of plants and local breeds of animals, including related traditional technologies^24^.

The total surface of Transylvania amounts to 100,293 km^2^ (42.1% of the total area of Romania), with a population of 7,221,733 inhabitants (approximately one third of the country's population). The relief of Transylvania is very varied. It includes: plains, hills, plateaus and mountains. The landscape of Transylvania is dominated by forests, pastures, agricultural lands, and vineyards.

During the communist period, 85% of Romania's agricultural areas were cooperativised. In these areas, the owners were dispossessed of their landed property by the communist state, following the Soviet model. Cooperative agriculture was an industrial one, practiced on large areas. After the fall of communism in 1989, the former landowners were re-owned. As many of the owners were no longer alive, the rights reverted to their descendants. Thus, Romania's agriculture was very fragmented, there were many small plots, scattered, with different owners. This greatly complicates the process of associating landowners for better management. In 2016, Romania owned over a third of the total number of agricultural holdings registered at EU level. According to the Statistical Yearbook of Romania (2017 and 2019), in 2016 about 91.6% of agricultural holdings in Romania had areas of less than 5 hectares, and the working population in agriculture was 23.1%. The average size of farms in the European Union in 2016 was 16.6 hectares, and the working population in agriculture was 4.5% (EUROSTAT). For the preservation of the genetic diversity of crop plants in Romania, these aspects represent an advantage, because landraces are suitable for cultivation on small areas, they are associated with traditional techniques that require a lot of manual labour. In Transylvania, the accentuated cultural diversity is an additional chance for the conservation of landraces.

### The questionnaire method

To obtain the information the method of the questionnaire with fixed questions with undisguised multiple-choice was used^25^. In this action were involved over 100 students, master students, doctoral students, researchers, academics and volunteers from three NGOs (Eco Ruralis, Eco Transylvania, Civitas), during the period 2007–2015.

The questionnaire includes 15 questions with 77 possible answers. All 338 small vegetable seed producers from whom landrace seeds were collected were interviewed. Therefore, the respondents were not selected based on age or gender; the only criterion of choice was the cultivation of landraces. Details about the respondents can be found in the Results chapter of this paper.

### The stages of identification and conservation of landraces

Our journeys aimed to both, obtaining the information and collecting seeds. These activities took place in the agri-food markets (26), in seed fairs (18) and gardeners’ farms (294).

Seeds were collected from 565 cultivars belonging to 26 species: artichoke (*Cynara cardunculus* L. subsp*. cardunculus*), pepper (*Capsicum annuum* L.), cucumbers (*Cucumis sativus* L.), from the *Brassica oleracea* complex the cauliflower (*Brassica oleracea* L. var. *botrytis* L.), white cabbage (*Brassica oleracea* L. var. *capitata* L.) and Brussels sprouts (*Brassica oleracea* L. var. *gemmifera* D.C.), pumpkin (*Cucurbita maxima* Duchesne), zucchini (*Cucurbita pepo* L. subsp. *pepo*), beans (*Phaseolus vulgaris* L.), lovage (*Levisticum officinale* W. D. J. Koch), red orach (*Atriplex hortensis* L.), dill (*Anethum graveolens* L. ssp. *hortorum* Alef.), pea (*Pisum sativum* L.), carrot (*Daucus carota* L. subsp. *sativus* (Hoffm.) Schubl. & Martens), ground cherry (*Physalis peruviana* L.), parsley (*Petroselinum crispum* (Mill) Fuss var. *radicosum* (Bernh.) Mart. Crov.), melon (*Cucumis melo* L.), watermelon (*Citrullus lanatus* (Thunb.) Matsum & Nakai, summer radish (*Raphanus sativus* L. var. *sativus*), winter radish (*Raphanus sativus* L. var. *niger* J. Kern), lettuce (*Lactuca sativa* L.), garden sorrel (*Rumex patientia* L.), tomato (*Solanum lycopersicum* L.), celery (*Apium graveolens* L. var. *rapaceum* (Mill.) DC) and eggplant (*Solanum melongena* L.). In order to identify the landraces, experimental fields were set up, where the morphological and agronomic characteristics of the plants were analysed. For the taxonomy and nomenclature of the collected crops, for each taxon the updated nomenclature based on GRIN Taxonomy for Plants was used. The observations made by us in the experimental field were correlated with the information obtained from the seed producer. The researchers' team included breeders with rich experience in the creation of vegetable varieties. For each authentic local variety that entered the BGS seed collection, passport data were recorded, based on FAO/IPGRI/Bioversity Multi-Crop Passport Descriptor Lists.

For landraces redistributed to seed houses all the information has been included in a single description field, much more simplified and accessible to the public concerned.

**Figure 1:**
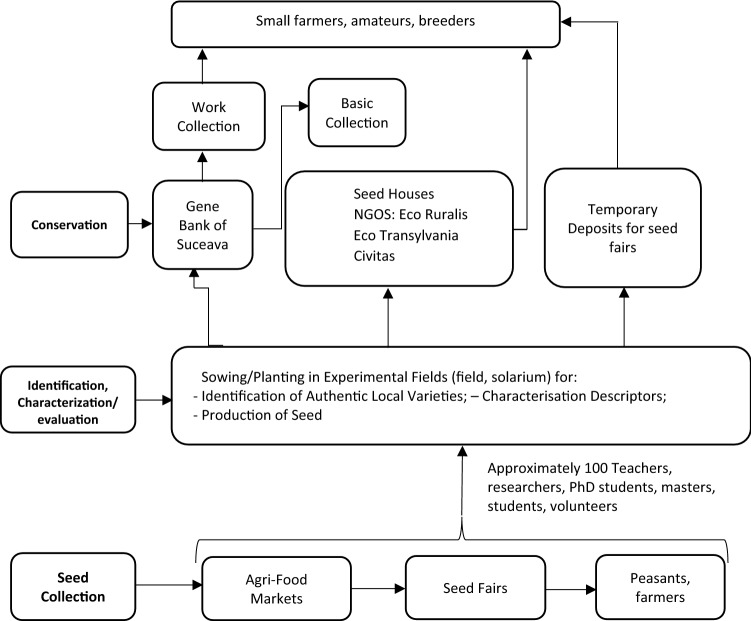
Operations scheme of collection, identification, characterization, seed production, redistribution and conservation of landraces in Transylvania, between 2007 and 2015.

The obtained seeds were preserved both in the Gene Bank of Suceava and in the seed houses of some NGOs. In the Gene Bank, the seeds are kept in both the work collection and the base collection. In seed houses, the genetic material is kept for shorter periods, the main purpose being to provide free seeds to farmers and to facilitate the exchange of seeds between peasants (Fig. 1).

Our research did not involve people under the age of 18. The need for informed consent was waived off by University Ethics Committee of the University of Agricultural Sciences and Veterinary Medicine Cluj Napoca (UEC-UASMV Cluj-Napoca). Also, our experiences did not include human experiences, nor did they work with human tissues. All methods were carried out in accordance with relevant guidelines and regulations. All experimental protocols were approved by UEC-UASMV Cluj-Napoca.

## Results

### The situation of landraces in Transylvania

In the period 2007–2015, seeds from 338 small seed producers were identified from all the counties of the historical province of Transylvania (except Caraş-Severin County). Seed producers originate from 210 localities, in particular villages (Table 1). The number of visited seed producers varies between 8 (Brasov County) and 41 (Cluj County).

**Table 1:** Data on seed collection from small producers of vegetable seed in Transylvania, from 2007 to 2015.

Crt. no	County (abbreviation)	The number of localities included in the study	Number of seed producers questioned	Number of species taken in the study	Cultivars		
					Taken into the study	Authentic landraces	
						No	%
1	Alba (AB)	12	28	20	46	27	58.7
2	Arad (AR)	11	17	9	29	9	31.0
3	Bihor (BH)	14	21	11	38	15	39.5
4	Bistrita-Nasaud (BN)	17	32	22	49	35	71.4
5	Cluj (CJ)	28	41	18	41	27	65.8
6	Brasov (BV)	5	8	6	32	21	65.6
7	Covasna (CV)	9	17	13	27	16	59.3
8	Harghita (HR)	15	22	21	33	23	69.7
9	Hunedoara (HD)	17	28	19	37	26	70.3
10	Maramures (MM)	14	22	26	62	47	75.8
11	Mures (MS)	12	18	16	34	15	44.1
12	Salaj (SJ)	20	33	25	47	25	53.2
13	Satu-Mare (SM)	16	24	16	39	12	30.8
14	Sibiu (SB)	11	14	18	28	12	42.9
15	Timis (TM)	9	13	7	23	6	26.1
Total/average		210	338	26	565	316	55.9

They were highlighted by high percentages of authentic local varieties, Maramures (75.8%), Bistrita-Nasaud (71.4%) and Hunedoara (70.3%) counties.

**Figure 2:**
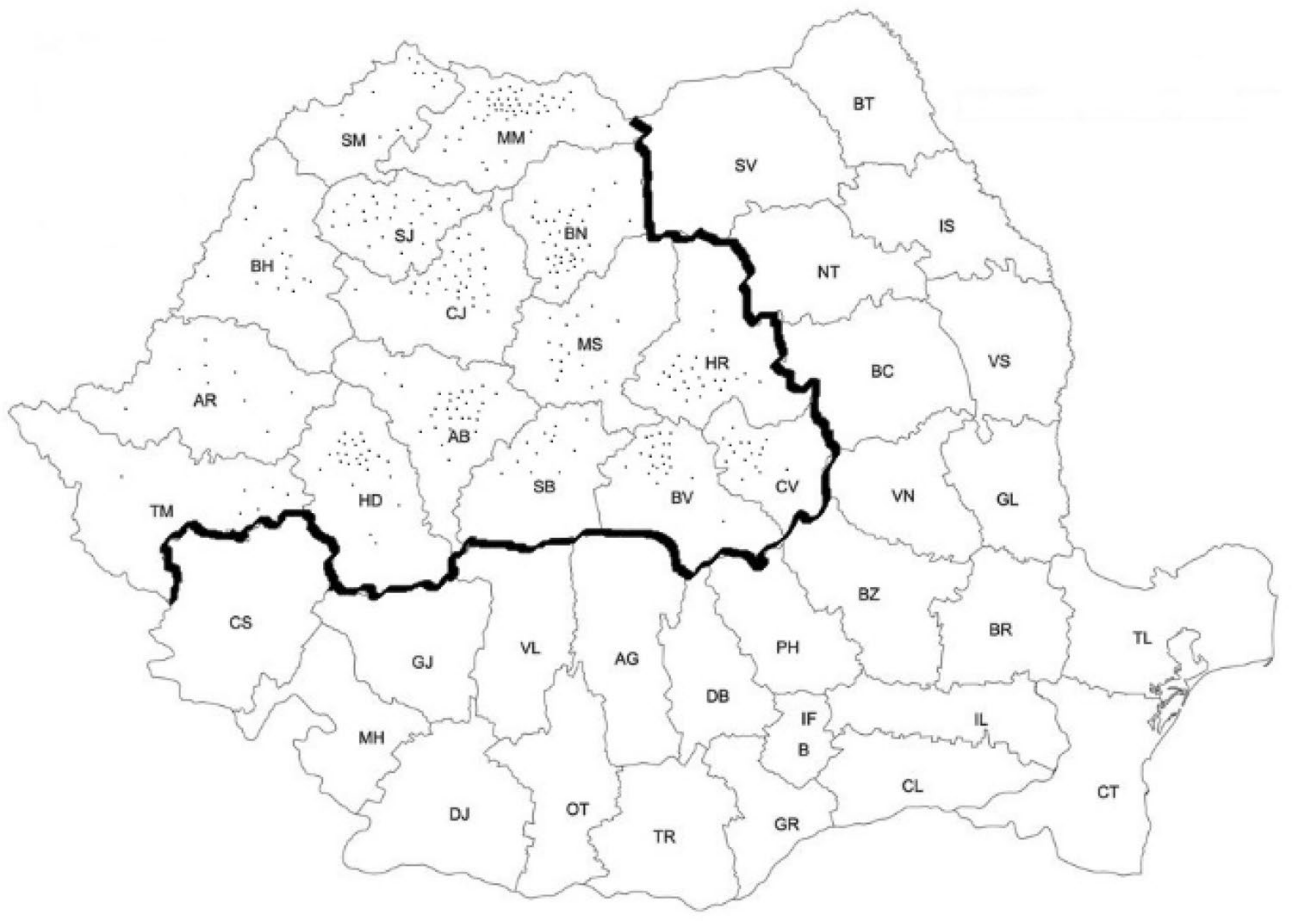
Distribution of identified local varieties of vegetables on the territory of Transylvania, during 2007–2015. The figure created using the Free and Open source QGIS version 3.4.0 (https://qgis.org/2019)^59^.

The data from Fig. 2 shows the geographical breakdown of the identified authentic local varieties. We note that the counties of Timis (TM), Arad (AR), Satu-Mare (SM) and the plain part of Bihor County (BH) are the poorest in traditional vegetable varieties. These areas are devoted to market gardening, and farmers prefer to use the modern varieties of crop plants, to the detriment of local populations. Here, market gardening is practiced in industrial systems and are the main source of income of farmers specialized in vegetable cultivation. Most market gardeners in these areas are not producers of vegetable seed.

The largest genetic diversity in vegetables we meet in hill and mountain areas (low altitudes), where market gardening represents only a secondary source of income. Each point on the map represents a local variety and is located in the area where it came from. In Salaj County we can see the most uniform distribution of authentic local varieties, given the predominant hill terrain.

Most landraces were registered in tomato (102), lettuce (101), carrot (85) and parsley (65). This also indicates the importance of these crops for farmers in the area taken into study. Basically, in Transylvania, there is no vegetable garden without these four species.

We note among the species where have been identified local varieties also for *Physalis peruviana*. This species has no tradition in Romania. It comes from tropical America and has begun to be cultivated in Transylvania only in the last two and a half decades. Peruvian ground-cherry has expanded into crop also here due to favourable ecological conditions, simple cultivation technology, multiple uses and the growing preference of Romanian consumers for its fruit. According to the farmers' declarations, the seed brought from abroad was from landraces, and our observations confirmed it. In the spontaneous flora of Romania only *Physalis alkekengi* is found, being used as a decorative plant and in phytotherapy. A similar situation is also at Brussels sprouts and cardoon which do not have a long tradition in the Romanian vegetable gardens. Therefore, *Physalis peruviana*, *Brassica oleracea* L. var. *gemmifera* and *Cynara cardunculus* we consider them to be local are new, secondary landraces having at least two decades of cultivation in Romania.

### Certification of tomato varieties originating in derived from landraces

In the year 2017 was approved by the State Institute for the Testing and Registration of Varieties (ISTIS) two tomato varieties from the most valuable landraces collected. It is about the varieties Cassiana and Danamari. Cassiana comes from the Marin village, Sălaj County, while Danamari originates in Sebeş-Alba, Alba County. Both varieties are suited to be grown either in the greenhouse or in the field. In addition to the special organoleptic qualities, the two varieties exhibit average blight resistance (*Phytophtora infestans*), making them pretable to be cultivated in ecological system.

It is important to note that for the cultivation of landraces in the ecological system, a derogation from the certification and inspection body is needed. Thus, a simple way to facilitate the introduction of local forms in the ecological cultivation system is to register these varieties in the Official Catalogue. Unfortunately, the procedures are quite complex and expensive, and not affordable to small-scale vegetable growers.

### Social and technological aspects related to the small vegetable producers in Transylvania

Table 2 presents several social and technological aspects related to small seed producers and the technologies they apply. This information explains to some extent the data previously presented in this work. Thus, we note that, of the 338 producers of vegetable seed, only 69 of them, i.e. 20.4%, are traditional producers. Most vegetable gardeners (29.9%) do the production of seed during two generations. This category might be considered an important one, since the fact that the second generation took over from the first this concern and did not abandon it may be a clue of the continuity of family tradition. However, the answers to the second question, related to the chances of passing to the next generations, are quite daunting. Only 37 (10.9%) of the 338 farmers interviewed are optimistic and extremely optimistic about this. Others believe that the chances are average (9.2%), small (48.5) and even null (31.4%).

**Table 2:** Socio-economic and technological aspects related to the production and conservation of local vegetable landraces in the Transylvanian area (Romania), between 2007 and 2015.

Crt. No.	Obtained information	Number	%
**Total seed producers questioned: 338**			
1	**Tradition in seed production**		
	a. Up to 5 years	23	6.8
	b. 5–15 years	60	17.8
	c. A generation	85	25.1
	d. Two generations	101	29.9
	e. More than two generations	69	20.4
2	**What are the chances of passing this tradition and these local varieties into the family?**		
	a. No chance	106	31.4
	b. Small chances	164	48.5
	c. Average chances	31	9.2
	d. High chances	13	3.8
	e. Safe chances	24	7.1
3	**Sex and age of seed producers**		
	a. Man	24	100
	a_1_ Under 40 years	12	50.0
	a_2_ 40–60 years	1	4.2
	a_3_ Over 60 years	11	45.8
	b. Women	314	100
	b_1_ Under 40 years	33	10.5
	b_2_ 40–60 years	61	19.4
	b_3_ Over 60 years	220	70.1
	c. Women and men	338	100
	b_1_ Under 40 years	45	13.3
	b_2_ 40–60 years	62	18.3
	b_3_ Over 60 years	231	68.4
4	**Seed source**		
	a. Own harvest	318	94.0
	b. Local community acquisitions	20	6.0
5	**The seed producer is also a creator of varieties**		
	a. Yes	2	0.6
	b. No	336	99.4
6	**The biological status of the samples collected**		
	a. Wild form	0	0
	b. Local variety	333	98.5
	c. Old breed	5	1.5
	d. Hybrid	0	0
7	**The surface on which the vegetable seed is produced**		
	a. 100–500 m^2^	88	26.0
	b. 500–1000 m^2^	196	58.0
	c. Over 1000 m^2^	54	16.0
8	**Disease and pest control in seed crops**		
	a. By making one treatment on average with organic pesticides	13	3.8
	b. By making two organic pesticide treatments on average	34	10.1
	c. By making on average three or more treatments with organic pesticides	84	24.9
	d. Only by using traditional methods of control without the use of organic pesticides	317	93.8
	e. Without phytosanitary treatments	21	6.2
9	**Seed production**		
	a. Annual plants		
	a_1_ Parcel for the production of seed	84	24.9
	a_2_ Ordinary parcel	254	75.1
	b. Biennial plants (in the 2nd year of vegetation)		
	b_1_ Parcel for the production of seed^a^	331	97.9
	b_2_ Ordinary parcel	7	2.1
10	**Risk of crossing varieties of the same species in seed plots**		
	a. Is known	333	98.5
	b. Is unknown	5	1.5
	c. The difference between the cross-pollinated and the self-pollinated species is made and measures are taken to prevent their crossbreed	289	85.5
	d. The risks are known but no distinction is made between the cross-pollinated and the self-pollinated species, and the measures to prevent their crossbreed are the same	98	29.0
	e. The distances provided in the legislation between parcels with different varieties of the same species	64	18.9
	f. Is taken into consideration this risk, but the distances they adopt (even if they do not comply with the regulations) is considered to be sufficient	274	81.1
	g. They take the necessary measures including to avoid cross-breeding with plants from spontaneous flora near seed crops (refers only to carrot seed producers)	72 from 85 carrot seed producers	84.7
11	**Selection of seed crops**		
	a. When planting propagating material (biennial plants)	338	100
	b. During the vegetation period (annual and biennial plants)	329	97.3
	c. When harvesting seeds	338	100
12	**Seed exchanges**		
	a. Only with farmers in the home locality, and the sale is occasional	98	29.0
	b. With farmers from home and other localities, and the sale is occasional	11	3.3
	c. No seed exchange or sale	74	21.9
	d. The seed is intended only for sale on the market	155	45.8
13	**The area where the seed is sold (refers to the 155 seed producers)**		
	a. In the home locality and in neighbouring localities	151	97.4
	b. In the home and other areas of the county	78	50.3
	c. Only in other counties of the country	4	2.6
14	**The number of seed buyers per year**		
	a. Under 50	18	11.6
	b. 50–100	31	20.0
	c. 100–200	42	27.1
	d. 200–300	54	34.8
	e. 300–400	10	6.5
	f. 400–500	0	0
15	**The production of vegetable seeds represents for the respondents**		
	a. The only source of income	0	0
	b. Important source of income	11	3.3
	c. Important source of income to which the production of vegetables is added	40	11.8
	d. Secondary source of income in addition to the production of vegetables	104	30.8
	e. Obtaining income from the sale of seeds only occasionally	35	10.4
	f. Just seed exchange (not selling seeds)	74	21.9

^a^Except for the parsley to which the seed is obtained from the ordinary parcel.

Question number 3 of the questionnaire develops the most worrying thing to maintain on the farm the local vegetable varieties in Transylvania. Thus, of the 338 seed producers 231 (68.4%) have the age over 60 years. If we corroborate this state of fact with the very low percentage of those who are optimistic about the continuation of this activity in the family, it means that in the coming decades the phenomenon of genetic erosion in vegetables can drastically accentuate accelerate in the area taken into the study. We also find that in Transylvania, the production of traditional seed is a specific concern of women (92.9%).

The seeds of the vegetable gardeners come from their own harvest, overwhelmingly majority (94%), while only 6% used the acquired seed within the local community.

Small seed producers are generally not creators of varieties. Only two of them (0.6%) seeks to obtain new varieties of crop plants without, however, intending to certificate them. Their motivation is not a material one, but it is about passion for plants and agriculture. One of them achieved encouraging results in tomatoes, and the other to carrot and lettuce. Other vegetable gardeners (99.4%) practice an empirical selection of the most valuable plants each year.

The biological status of the collected samples is: local varieties (98.5%) and breeds (1.5%). The vegetable gardeners interviewed do not use in their farms wild forms from any species. We can see that local varieties hold the weight. The breeds used have been purchased for more than 15 years, so that morphological and production characteristics have changed greatly from the original material.

The areas intended for the production of vegetable seed range from 500 to 1000 m^2^ to 58% of respondents, respectively between 100 and 500 m^2^ to 26% of them. Only 54 of the 338 seed producers cultivate areas of more than 1000 m^2^ for seed production.

Local variety holders make plant protection treatments only in some species and in some parasites that can compromise the crop, such as: at cucumbers for angular leaf spot (*Pseudomonas lachrymans*) and blight (*Pseudoperonospora cubensis*), in tomato for blight (*Phytophtora infestans*), the eggplant for the Colorado beetle *(Leptinotarsa decemlineata)* and the white cabbage for the black flea of the cruciferous or cabbage flea beetle *(Phyllotreta atra)*, the cabbage moth (*Mamestra brassicae*) and the cabbage white butterfly (*Pieris brassicae*). Against the other parasites, the sprinters with pesticides are made in a completely exceptional way, only in some years. It is encouraging that 93.8% of the seed producers use traditional methods of control of diseases and pests: agrotechnical methods, macerated of plants (especially nettle and horsetail), manual collection of some pests, etc. The 21 seed producers who do not carry out any phytosanitary treatment pursue a most rigorous selection of disease and pest resistant plants, at the risk of total loss of harvest in some years. They practice organic farming or intend to move to this farming system, where the genetic resistance of plants to parasites is a fundamental element.

At the annual plants, most of the vegetable gardeners (75.1%) produce the seed in the production plots where the plants are maintained to continue to obtain seed. At the biennial plants, the planting stock is planted in special plots for the production of seed, to 97.9% of those interviewed. The exception makes those who produce the seed of parsley where the roots are kept still over the winter in the field, under a layer of straw or corncobs, to be protected from frost. The vast majority of seed producers (98.5%) are aware of the risks related to the crossing of varieties from the same species. We mention that the 5 producers who do not know this aspect are beginners in this activity. A significant proportion of the surveyed vegetable gardeners (85.5%) make the difference between the cross-fertilised and autogamous species, however in a similar percentage (81.1%) does not comply with the legal regulations regarding the distances between varieties of the same species. They appreciate the necessary distances between varieties and are oriented according to the possibilities and the way they organise their vegetable garden. A similar percentage (84.7%) of carrot seed producers take the necessary measures so that the farmed carrot does not cross with the wild carrot which is very present in the spontaneous flora of Transylvania.

The selection of seed-producing plants is made 100%, both in planting (in the case of biennial plants) and in seed harvesting. Producers say that the seed is harvested only from the most vigorous and productive plants. The selection is also made during the vegetation period by the vast majority of the vegetable gardeners (97.3%).

The exchange in seeds is an old practice in the Transylvanian market gardening and it led to the infusions of genes that made possible the appearance of valuable local varieties. These exchanges are also made today but, on a much smaller scale. Most seed exchanges are carried out between the same community members (18.6%), 10.4% among the extended family members, and 3.3% with vegetable gardeners from other localities.

Almost half of the vegetable gardeners (48.5%) use the seed for sale on the free market. Most sales are made in the residential locality and neighbouring localities (97.4%), on the days when the market is held. In Transylvania, most localities centres have a day of the week when peasants and small farmers come to sell their agricultural and animal products. Most small seed producers have between 100 and 300 buyers per year. None of the respondents reported more than 400 seed buyers per year. The explanations come from the answers to the next question from which we learn that none of small farmers does not have as their sole source of income the production of seed. The vast majority of them obtain the income also from the sale of vegetables.

## Discussions

Romania has an important potential of agricultural development which is underutilized. With 61.3% (14.6 million hectares) of the country area used as agricultural land, 23.7% of Romania's population works in agriculture (EUROSTAT). This category includes employees from farms, day workers, small households and farmers who have submitted applications for subsidies. According to the Statistical Yearbook of Romania (2016), the areas cultivated with vegetables occupy only 1.56% of the agricultural area, and from these, the most cultivated are cabbage, tomatoes, onions and peppers. However, the genetic diversity in vegetables is still a rich one, as it is preserved mainly in the home gardens. Basically, in Romania, it is almost impossible to find household in rural areas without a vegetable garden that ensures a good part of the family's vegetable needs. Both the high percentage of the population working in agriculture and the cultivation of local vegetable varieties in family gardens are important conditions for reducing the phenomenon of genetic erosion. Just like us, many researchers consider the family gardens an important opportunity for the conservation on farm of landraces in vegetables:^13,19,26,27^. Montesano et al.^28^ explains the smaller genetic erosion of vegetables, compared to grain just by very frequent cultivation of vegetables in the home gardens. In the Basilicata region of Italy, the same authors propose public economic support for the "farmer-maintainers" of landraces.

The on farm conservation of vegetable landraces is an extremely important approach because each year, through natural and anthropic selections, varieties are evolving and continuously adapting to climate change. This type of dynamic conservation is, in many cases, associated with traditional crop techniques, where productions are much lower compared to industrialised agricultural systems. That's why it is desirable growers to be stimulated to favour in cultivation and maintenance of these landraces, through granting compensatory payments adopting adequate European, national or local policies. Although in some European countries such measures have been implemented, in Romania, the National Rural Development Plan (NRDP) 2014–2020 does not provide any facilities to farmers who want to cultivate landraces; by Measure 10 of the NRDP (Agri-Environment and climate), compensatory payments are granted only to farmers who breed endangered animals. This situation is also found in other European Union countries^13^. On the other hand, in Italy, the administration of the Puglia region provided financial support for five years to farmers who maintain plant genetic resources in situ. The measures also covered the creation of a network of 'seed rescuers'. These actions were conducted within the Rural Development Programme 2007–2013^19^.

The Implementation of the European directives in Romanian legislation contributed to the valorization of landraces. Thus, by Law No. 266/2002 with subsequent amendments, it is allowed to trade on local markets in Romania, small quantities of seed of non-registered breeds in the Official Catalogue. The condition is to be *without prejudice to phytosanitary quarantine regulations and to ensure their quality*. Furthermore, in organic farming, the certification and inspection body may grant derogation for the use of uncertified seed of landraces (EC Regulation no 889/2008, art. 45). About 40% of organic operators in Transylvania's agriculture use local varieties in the own farms, which is beneficial and encouraging for their preservation.

Although Romania ratified, in the year 2005, the International Treaty on Plant Genetic Resources for Food and Agriculture and transposed the European Union legislation into national law, local varieties of crop plants did not reach into the attention of decision-making institutions at central and local levels. Thus, the State Institute for Testing and Registration of Breeds in Romania (ISTIS) registers in the Official Catalogue only the varieties that comply with the DUS criteria (distinction, uniformity, stability). On the other hand, the varieties previously registered, but at which the author has given up and the seeds are no longer officially maintained, appear in the Catalogue as "breeds in conservation". So, "conservation breeds" and not "conservation varieties". From here, it resulted the error that we find in some publications claiming that associations of farmers and private citizens from Romania managed to record 15 conservation varieties of wheat, oats, grain and barley in the Official Catalogue of breeds. Until present, two tomato landraces were certified as conservation varieties (Cassiana and Danamari), by the University of Agricultural Sciences and Veterinary Medicine Cluj-Napoca, in the year 2017. These breeds have gone through all the stages, formalities and rigor of testing and approval of ISTIS (2017)^29^. The authors recommend the use of the two varieties in organic crop, given the average resistance to blight (*Phytophtora infestans*) and the exceptional taste qualities. In other countries such as Finland the rules for registering landraces in the National Catalogue are less restrictive^30^. In Italy, regional laws promote valuable traditional varieties by registering them in the regional registers of varieties^15^.

An effective conservation strategy for plant genetic resources cannot be achieved without the national inventory of conservation varieties. In addition, to this inventory, it is important to identify the existing vulnerabilities in this field. This is the opinion of the majority of researchers concerned about the conservation of biological diversity in the agricultural field, and our investigations and researches fall within this optics^26,31–39^.

The method of the interview directly applied by us proved to be very effective. Seeds from small vegetable gardeners were also collected on this occasion. In other areas, researchers also appealed to the phone interview but, under our conditions, this method did not work^35^. We believe that, in direct interviews, respondents are much more open, truthful and more generous with answers to questions. In addition, the second objective of our expeditions was the collection of seed in order to establish experimental fields for the identification of authentic landraces and seed production. Where possible, we have made a preliminary morphological and agronomic characterization even in the place of provenance of the collected local variety. Interviews similar to ours have been successfully used by Balyejusa et al.^40^ and Montesano et al.^28^. Balyejusa et al. choose to interview 3–17 farmers to obtain information about the preferences, utilization and naming of their most commonly grown varieties. Subsequently, they chose only one farmer from each village for more in-depth interviews. We conducted in-depth interviews with all respondents. In the Basilicata region of Italy, Montesano et al. interviewed all farmers from whom they harvest seeds, just as we did. These questions relate to their age, family structure, various information about the farm and the landraces they own, and the context in which the landraces were preserved.

In Romania, the three regions with a special interest for the on-farm conservation of local varieties are Maramures (MM), Suceava (SV) and the Apuseni Mountains^23,41^. It confirms, basically, the results of our investigations. To those found by the authors of the said work—which operates at the Bank of Plant Genetic Resources Suceava (BRGVS)—we can also add the Intra-Carpathian corners from the Eastern and Southern parts, especially the counties: Bistrita-Nasaud (BN), Harghita (HG), Brasov (BV) and Hunedoara (HD).

In Romania, the largest genetic diversity in crop plants is in beans, maize and potato^23^. This remark is explained by the fact that BRGVS started its activity as a plant genetic resources laboratory within Suceava Agricultural Research Station. Therefore, our investigations allow us to add to the list of species with high genetic diversity also the tomato (*Solanum lycopersicum*), lettuce (*Lactuca sativa*), carrot (*Daucus carota*) and parsley (*Petroselinum sativum*). Most likely, future research could extend this list^42–44^.

In our actions, the identification of landraces was made on the basis of the morphological and agronomic characteristics of the plants to which the information obtained from the seed producers was added. On these criteria did Sudré et al.^45^ the assessment of genetic diversity at *Capsicum* spp, Boros et al.^46^ to beans, Sabatino et al.^47^ at bottle gourd, D'anna and Sabatino^48^ at eggplant, Dersouni and Chougui ^49^ to the tomatoes. Casals et al.^50^ highlights the importance of phenotypic characteristics of fruits to two breeds of tomatoes in Spain (Montserrat and Pera Girona) which have different, highly profitable market niches. Although the molecular analyses (Amplified Fragment Length Polymorfirm—AFLP) showed very small differences between the two varieties (8.5% of polymorphic loci), phenotypical they exhibit very high differences in the morphology of the fruit.

The fact that 93.8% of our respondents use traditional methods of controlling parasites in plants, without the use of chemical pesticides, confirm the fact that landraces can contribute to the application of environmentally friendly crop technologies. Gibson et al.^51^, Onyeka et al.^52^, Sarker and Erskine ^53^, El Tahir ^54^, Esquinas-Alcázar ^55^, Newton et al.^56^, Ceccarelli ^57^, Ceccarelli ^58^ and others, also reached these conclusions.

A real danger to local varieties is the old age of seed producers and vegetable gardeners using these seeds. Seed producers that have been the subject of our investigations show that 68.4% of them are over 60 years old and only 13.3% are under 40 years old. It is interesting that 93% of traditional seed holders are women and only 17% men. In Transylvania, the proportion of young people under 40 years old is slightly higher than in the Basilicata Region of Italy, where it represents only 9%^28^. Instead, in the same region, the farmers interviewed who are over 60 years old are about half of the owners of germplasm. If in Transylvania, the production of seed is categorically in favour of women (93%), in the region of Basilicata the report is in favour of men; however, the ratio between the two sexes is much more balanced (45% women, 55% men). In another region of Italy (Puglia), the risk of losing the landraces of carrot is worrying because of the cultivation on small surfaces and the age of the local farmers (60–75 years)^19^. Studies made on the territory of Italy by Veteläinen et al.^13^ show that the average age of farmers conserving landraces on farm is 60 years old^13^.

## Conclusions

Romania still has a rich genetic diversity of vegetables, especially in the hill areas that not passed the cooperativization process during the communist period. In these areas, traditional, family-based agriculture continued to maintain after the emergence of industrialised agriculture.

In Romania, as in other countries, landraces are subject to the risk of extinction. The biggest danger lies in the old-age of small vegetable seed producers (68.4% over 60 years old) and the lack of interest of the majority of their followers for technologies and old varieties.

Increasing efficiency in the conservation of landraces in Transylvania, but also in Romania, in general, can be done by: granting compensatory payments to farmers using landraces through the National Rural Development Plan which will be built on the next CAP (2021–2027); setting up annexes to the official Catalogue of varieties and reducing the requirements for landraces introduced in these annexes; expanding organic farming and maintaining the possibilities to use the local populations in these technologies; stimulating NGOs carrying out activities in the identification, collection and conservation of traditional seeds; encouraging seed houses and seed fairs which facilitate the exchanges of seed in landraces; population awareness of the importance of this genetic heritage and the benefits of its conservation and enhancement.
